# Atmospheric Emissions, Depositions, and Transformations of Arsenic in Natural Ecosystem in Finland

**DOI:** 10.1100/tsw.2002.822

**Published:** 2002-02-22

**Authors:** Arun B. Mukherjee, Prosun Bhattacharya

**Affiliations:** Department of Limnology and Environmental Protection, P.O. Box 62, FIN-00014 University of Helsinki, Finland; Division of Land and Water Resources, Royal Institute of Technology, SE-100 44 Stockholm, Sweden

**Keywords:** arsenic, emissions, groundwater, mechanisms, Finland

## Abstract

For the last 2 decades, special attention has been paid to arsenic due to its high concentration in groundwater in many regions of the globe. There are not very many reports on arsenic concentration in the Finnish ecosystem, although the metal has been known to be highly toxic since ancient times. For the majority of people in Finland, the leading exposure route to arsenic is through food consumption.

In this study, it has been observed that atmospheric emissions of arsenic from anthropogenic sources have decreased by 90%, which is due to better control technology and strict regulation. Aquatic discharge also was attenuated from 7.1 metric tons (t) in 1990 to 0.7 t in 1999. The concentration of arsenic aerosols in the atmosphere in Finland varies between 0.46 to 0.75 ng m^–3^. Its use in pesticides and insecticides also has been phased out in Finland. There is no information available regarding arsenic species in the Finnish environment.

Elevated concentrations of arsenic in groundwater has been reported for many countries. In Finland two hot spots are reported – one in the south of Finland and the second in Lapland. In these areas, arsenic concentration in well water is greater than 10 μg l^–1^ (WHO recommended value: <10 μg l^–1^). It is believed that the release of arsenic into the Finnish groundwater is geogenic.

## INTRODUCTION

There is very little historical information available regarding arsenic released in Finland or in Europe. Most studies focus on lead, cadmium, and mercury and fewer on arsenic, copper, chromium, nickel, vanadium, selenium, or zinc[1]. But, this does not mean that estimation of anthropogenic flux of these latter elements has been ignored completely. Arsenic is toxic and ubiquitous in the environment. At the end of the last millennium, the **trained in [trend?]** atmospheric releases of trace metals including arsenic showed a substantial decrease in Finland and the European region. In Europe, atmospheric emission of arsenic decreased from 6,378 t in 1979 to 2,575 t at the beginning of the 1990s[2,3]. Accurate inventories of trace metals and their sources to the ecosystem are necessary to enable assessments to be made of the extent of national, regional, or global contamination and of damage due to occurrence of trace metals in the environment. This information is also useful to policy makers and the public (1) to formulate environmental agreements on the national or global scale, (2) to evaluate the environmental costs and benefits of different policies, and also (3) to identify the activities that are responsible for environmental damage.

Estimation of atmospheric flux of trace metals is generally based on emissions measurement, emission factors, and socioeconomic activities. The purpose of this study is (1) to find out atmospheric emissions and emission factors of arsenic, (2) to determine emission trends, and also (3) to find out to what extent natural release of arsenic in the Finnish groundwater has posed any threat and its release mechanisms.

## METHODOLOGY

In this study, atmospheric emission data for arsenic are based on the works carried out for trace metals emission factors and emissions by the Finnish Environment Institute in the last millennium. Arsenic emission data for 1999 were calculated in this study. Aquatic discharge of arsenic is based on the information registered in the database of the Finnish Environment Institute. Groundwater arsenic concentration in Finland was collected also from the Geological Research Centre of Finland. Today however, emission of arsenic, process information, abatement technology, raw materials used, and measurement methods are generally reported at the beginning of each year by the facility concerned and these are registered in VAHTI, the database of the Finnish Environment Institute. Figure 1 illustrates the flow of information on which emission factors and atmospheric arsenic emissions are based. The following formula was selected for calculating the arsenic emission.

$$E_{arsenic} = \_\, A_{activity} \times [\, \_\, F_{technology,\ arsenic} \times (P_{technology})] \qquad \text{activities}$$

$_{\text{technology}}$ **[pieces missing?]**

where $E_{arsenic}$ = emission of arsenic under study, $A_{activity}$ = activity rate for each activity, $F_{technology,\ arsenic}$ = emission factor for arsenic, $P_{technology}$ = penetration of pollutant through gas cleaning equipments.

## RESULT AND DISCUSSION

### Sources of Arsenic

There are two sources, natural and anthropogenic, by which arsenic enters into the atmosphere.

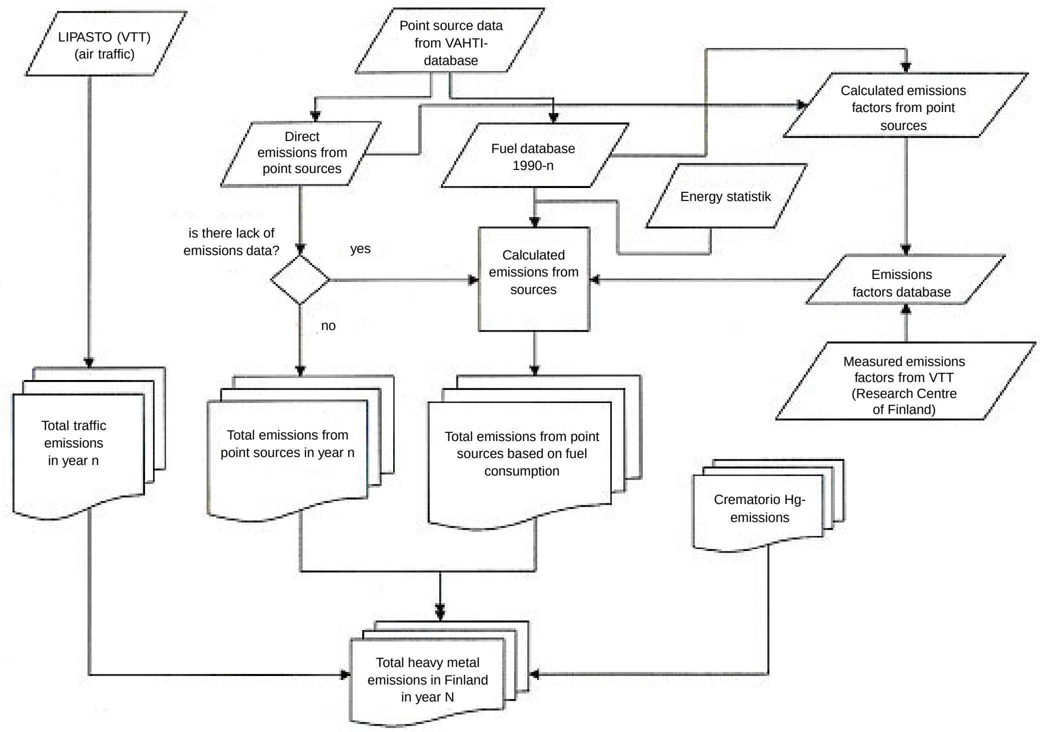

**FIGURE 1.** [caption?]

## *Natural*

In Finland, there is scarcity of data for natural emissions of metals, in spite of the fact that natural emissions of trace metals may be important on a national or regional basis. Nriagu[4] estimated that 30 to 50% of total global atmospheric emissions of arsenic stemmed from natural sources.

## *Industrial*

Arsenic emissions from industrial sources include utility boilers, various high-temperature industrial processes, waste incineration, and vehicle traffic. Arsenic-based fertilizers, pesticides, and wood preservers also create environmental problems. In the closing millennium, arsenic emissions decreased notably as a result of efficient particulate separation from gas streams, new process development, and better abatement technology[5,6].

### *Heat and Power Generation*

In Finland, total heat and power generation increased from 417,270 TJ (tera joule = $10^{12}$ J) in 1990 to 515,807 TJ in 2000 of which 11% was from coal burning in 1999[7]. Coal and crude oil are imported, and the country has a long history of peat use with the largest proportion of wetlands of any country of the world. The trace metals in coal have a tendency to associate with either the organic or inorganic fraction of coal. In the case of arsenic, it is mainly associated with pyrite or with other sulphide minerals[8]. Arsenic concentration in imported coal in Finland varies between 3.5 to 4 mg $kg^{-1}$, but occasionally higher values (14.4 mg $kg^{-1}$) have been reported[9]. In domestic peat, the arsenic concentration is about 2.2 mg $kg^{-1}$, but in fuel oil the concentration of arsenic is about 5.0 mg $kg^{-1}$[10].

In the sections of public power generation facilities, district heating, or industrial combustion, the basic mechanisms of trace metal emissions to the atmosphere are similar. Emission is generally based on fuel input quantities, the fuel qualities, the nature of the combustion installations, and air pollution control equipment. The melting point of arsenic is 817°C and it sublimes at 613°C. In the combustion plants, arsenic tends also to condense, as well as volatilise, onto the surface of smaller particulate in the flue gas stream. Estimated arsenic emission for the 1990s is given in Table 1 and selected emission factors for arsenic are shown in Table 2, for the interest of readers.

## *Oil-Fired Boilers*

Fly ash removal units are not generally necessary on oil-fired boilers. Both heavy and light quality oils are used in boilers; the concentration of arsenic is higher in the former than in the latter.

## *Peat and Biomass-Fired Plants*

Consumption of peat in Finland was about $24.5 \times 10^6$ $m^3$ in 1999 (90% milled peat and 10% sod peat). Its share in heat and power generation was about 6%. Bubbling fludized bed (BFB) and circulation fludized bed (CFB) systems are generally used in peat combustion plants, in which the combustion temperature is lower (1,100K) than in conventional, pulverised solid fuel-fired power plants (1,650K).

Biomass such as peat, bark, saw dust, and tree branches are used in BFB and CFB systems for heat and power generation. In 1999, about 14% of the total heat and power was produced from these and other similar raw materials. There is scarcity of information regarding the arsenic concentration in bark and tree branches, but one study indicates that arsenic concentration is about <0.1 mg $kg^{-1}$ in dry matter[11]. Due to lower operating temperatures in these combustion processes and the lower concentration of arsenic in raw materials, less emission of arsenic is expected from these sources.

**TABLE 1**
**Atmospheric Emissions (kg $a^{-1}$) of Arsenic in Finland, 1990–1999[5]**

| Source | 1990 | 1991 | 1992 | 1993 | 1994 | 1995 | 1996 | 1997 | 1999* |
|---|---|---|---|---|---|---|---|---|---|
| Energy and transformation industries | 779 | 679 | 633 | 394 | 904 | 555 | 721 | 65 | 496 |
| Nonindustrial combustion plant | 1,760 | 602 | 96 | 79 | 87 | 105 | 493 | 255 | 99 |
| Combustion in manufacturing industry | 414 | 504 | 497 | 424 | 416 | 323 | 374 | 304 | 1,300 |
| Process industry | 30,210 | 20,257 | 14,772 | 13,431 | 7,904 | 2,482 | 5,605 | 11,050 | 1,761 |
| Waste treatment and disposal | 0 | 0 | 0 | 8 | 37 | 19 | 2 | 2 | 3 |
| **Total** | **33,160** | **22,000** | **16,000** | **14,300** | **9,300** | **3,500** | **7,200** | **12,300** | **3,659** |
| | **33,163** | **22,042** | **15,998** | **14,336** | **9,348** | **3,484** | **7,195** | **11,676** | **3,659** |

*Calculated in this study.

**[[12,300 ok?]]**

**TABLE 2**
**Average Emission Factor (µg/MJ) for Arsenic from Combustion of Fossil Fuels as per the Database of the Finnish Environmental Administration[5]**

| Thermal Capacity (MW) | Techniques | Abatement Technology | Emission Factor (µg/MJ) |
|---|---|---|---|
| **Coal utility boilers** | | | |
| 300 | Mixed burner | a + b + c + d | 5.24 |
| 300 | Mixed burner | a + c + d + e | 0.61 |
| 300 | Jet burner | f + d | 0.43 |
| 50-300 | Mixed burner | d | 6.24 |
| **Heavy crude oil utility boilers** | | | |
| 50-300 | Other types of firing | Not specified | 2.09 |
| 50-300 | Grate firing & Pres. oil atomizer | Not specified | 8.62 |
| **Industrial boilers** | | | |
| 50-300 | Pres. oil atomizer | b + g | 9.85 |
| **Peat utility boilers** | | | |
| ??? | Circulating fluidized bed | h + i + g + d | 3.96 |
| 50-300 | Bubbling fluidised bed | j + g | 6.76 |
| **Wood-fired boilers/industrial boiler** | | | |
| 50-300 | Bubbling fluidised bed | Not specified | 2.62 |

*Note:* a, fabric filter; b, low-nox burner; c, semi-dry; d, electrostatic precipitator; e, Overfire air; f, wet scrubber; g, cyclone; h, feeding absorber to burning chamber; i, re-burning; j, other combustion air.

### *Metallurgical Industry*

In Finland, there are four important nonferrous metallurgical industries where copper, nickel, zinc, ferrochromium, and cobalt are produced from concentrates, ores, and scrap. In Outokumpu Harjavalta Metal Works, arsenic recovery was discarded in 1992 due to the falling demand, high toxicity, and strict environmental regulations. The imported copper concentrate contains 0.25% arsenic. However, as a result of improved technology, dust emissions from the copper and nickel smelters at Harjavalta Metal Works decreased from 1,100 t in 1985 to 48 t in 1999 (limit value: 90 t year$^{-1}$; Fig. 2; personal communication, Outokumpu Oyj, 2000). It means arsenic emission also decreased from the nonferrous metallurgical industry in Finland (Table 1).

### *Waste Incineration*

In Finland about 90% of wastes are normally disposed in landfills and the rest by incineration, composting, and recycling. There is only one incineration plant where 50,000 t of household wastes are burnt annually and in which energy is recovered for district heating. This plant is equipped with electrostatic precipitator (ESP), flue gas desulphurisation (FGD) system, and fabric filter (FF). The release of trace metals during incineration of wastes does not depend as much on the concentration in the waste itself, as on the chemical mechanisms acting at high temperature inside the reactor[12]. The emitted metals are often concentrated on the particles of diameter less than 1 µm in the waste gas. The entrained quantity of metal is a function of the size, shape, and density of the ash particles as well as the operating temperature of the incinerator[13]. However, the incinerator in Finland is equipped with the FF at the end of the gas stream, hence it is quite possible that atmospheric emissions of arsenic will be minimal.

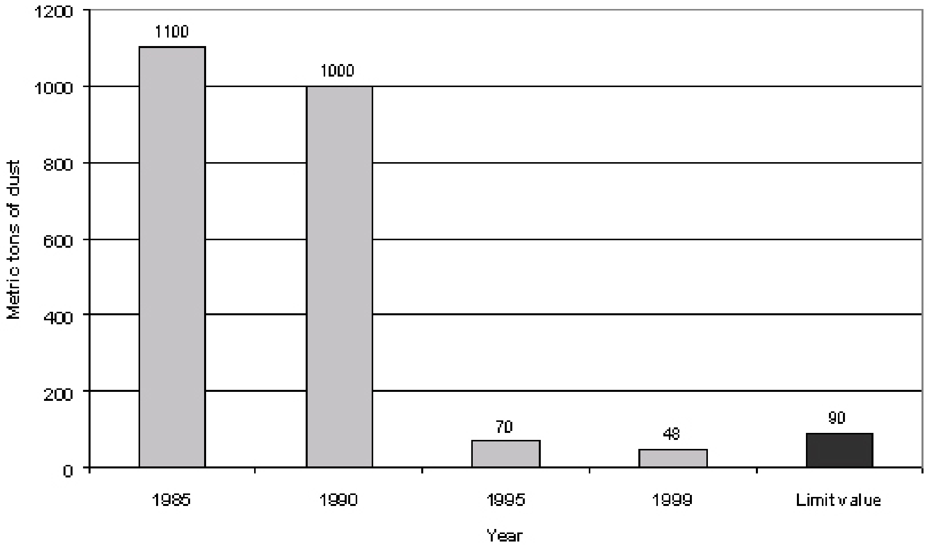

**FIGURE 2**. Dust emissions to the atmosphere from copper smelter in Finland.

## Arsenic from Other Sources

Information is available regarding aquatic release of arsenic to waterbodies in Finland. It is noted that processing industries still release the major amount of arsenic to the aquatic environment. As a result of strict regulations and better effluent treatment plants, the aquatic release of arsenic decreased from 7,130 kg in 1990 to 705 kg in 1999.

Arsenic and its compounds are no longer used in pesticides in Finland. In 2000, 510 t of arsenic pentoxides were used in Finland of which 320 t were imported for the production of copper-chrome-arsenate (CCA) chemical for wood preservation. Of the 2,100 t of CCA manufactured, 780 t were exported[14]. This chemical and other arsenic-based compounds used in the wood preservation industry have caused widespread contamination of soils and aquatic environment in many regions of the world[15,16]. The main reason for the universal use of this chemical as a wood preservative is the biocidic characteristics of $Cu^{II}$ and $As^{V}$**.** Chromium is used to bind arsenic and copper into cellular structure of the wood. Fixation of CCA is dependent on conversion of $Cr^{VI}$ to $Cr^{III}$. However, the arsenic in CCA is most mobile and toxic to the environment[17].

In Finland, Turpeinen et al.[18] studied speciation, mobilisation, and toxicity of arsenic in soil samples (sandy loam) near a wood impregnating factory in Lammi, southern Finland. Soil arsenic (total) concentration varied from 6,928 to 9,119 mg $kg^{-1}$, but the bioavailablity of arsenic and toxicity of As(V) and DMAA (dimethyl arsenic acid) for microbes were very low. It is believed that the soil bound the major part of the arsenic strongly due to its low pH (4.8–4.9) and high clay content. But microbial activities may change speciations of arsenic in soils as a result of which the toxicity, mobility, and reactivity of arsenic may be altered[19].

## CONCENTRATION OF ARSENIC IN THE ATMOSPHERE OF FINLAND

In the atmosphere, arsenic can exist as gaseous arsines, which are highly toxic to humans, and are generally formed in an anaerobic-reducing environment[18]. The arsenic in aerosols is important from the human health aspect. Aerosols of diameter <1 µm are of major importance, as these can be carried for a long distance and can enter into lungs during breathing. There are some values available regarding arsenic aerosols (fine + coarse) in the Finnish atmosphere and these are as follows (ng $m^{-3}$): Virolahti, southeast Finland, 0.75; Utö, south Finland, 0.59, and Ähtäri, southwest Finland, 0.46[6]. Jalkanen[6] observed a lower value of arsenic aerosols in Lapland in comparison to values in western Finland. In addition to particle size, knowledge of chemical speciation of arsenic is an important criteria for evaluating the toxicity of arsenic in the ecosystem[18].

Atmospheric pollutants are transported within air masses. The two Estonian, oil shale–fuelled power plants and cement plant in Kunda emit huge amounts of inorganic and alkaline particulates (arsenic emission: 8.9 t in 1992–1993 and dust emission: 135,500 t in 1993)[20,21] which have a pronounced effect on the presence of trace metals in the atmosphere of Finland.

Reports are available on trace metals deposition in the Nordic countries based on moss technique. In Finland, arsenic concentration in mosses varies between 0.2 to 0.6 µg $g^{-1}$ and decrease in the south to north direction[22].

## ARSENIC IN THE GROUNDWATER OF FINLAND

Groundwater is an important source for drinking water for a major part of the population in the developing and developed countries. In Finland, about 20% of the population is dependent on drilled well water. For the last 2 decades, arsenic found in groundwater has raised a serious problem in both developed and developing countries.

Natural mineral deposits containing arsenic may cause elevated levels of arsenic in groundwater. In Finland, bedrock and soils contain between 0.06 to 1.6 µg $l^{-1}$arsenic, and there are many areas in Finland which are prone to high concentrations of arsenic[23]. This is not alarming when compared to concentrations found in Bangladesh, West Bengal, India, Taiwan, and sedimentary aquifers elsewhere in the world. Soils and underlying bedrocks and their interaction with the aqueous phase play an important role in controlling the retention or mobility of arsenic under different redox conditions within the subsurface environment[24]. The important arsenic minerals in Finland are arsenopyrite (FeAsS) and, to a lesser extent lollingite ($FeAsS_2$). In central Lapland and southwest Finland (near Tampere), high concentrations of arsenic (0.138 mg $l^{-1}$) in drilled well waters has been reported which is geogenic. In general, arsenic concentration in the groundwater of Finland is controlled by the lithology of the bedrock. Its concentrations in groundwater were highest in wells drilled in terrains with mafic and ultramafic plutonic bedrocks (1.04 mg $l^{-1}$) followed by granitods (0.57 mg $l^{-1}$) and metasediments (0.484 mg $l^{-1}$)[25]. In the domain of sulfide mineralization, primary sulphide minerals oxidation in the bedrock is the prevalent mechanism for the mobilization of arsenic in the Finnish groundwater.

## CONCLUSIONS

In this survey, we have presented the scenarios of arsenic in Finland. Arsenic enters the atmosphere from natural and anthropogenic sources. There is very little information on natural emissions of arsenic for Finland, but studies show that atmospheric emissions from industrial sources decreased from 30.1 t in 1990 to 3.66 t in 1999. This decreasing trend is due to better control technology, strict regulations, and process development. The aquatic discharge of arsenic also decreased from 7.0 t in 1990 to 0.7 t in 1999. The process industry continues to release the largest amount, although particulate release from the nonferrous metallurgical industry decreased from 1,100 t in 1985 to 45 t in 1999. For this study, no information could be found regarding arsenic speciations released from high-temperature processes in Finland. There is information available on arsenic aerosols in the atmosphere of Finland, which varies between 0.46 to 0.75 ng $m^{-3}$, and in Lapland it will be still less. There is no information on long-range transport of arsenic in Finland, but huge amounts of alkaline aerosols from the power plants in Estonia are known to enter the atmosphere of Finland.

About 20% of the Finnish population is dependent on groundwater. There are two hot spots — one in Lapland and the other one near Tampere, where the arsenic concentration is >10 µg $l^{-1}$ (WHO recommended value: <10 µg $l^{-1}$). Arsenic in the groundwater in Finland is geogenic and its mobilisation is due to oxidation of minerals in the bedrock of the aquifers.

## ACKNOWLEDGEMENT

This paper was presented at the International Conference on the Biogeochemistry of Trace Elements, Guelph, Ontario, Canada, 29th July – 2nd August 2001. Many thanks to Dr. Ron Zevenhoven, Helsinki University of Technology and other anonymous reviewers for their thoughtful, critical comments. Language was corrected by Ken Himsworth and the assistance received in preparation of this manuscript from the staff members of the Department of Limnology and Environmental protection, University of Helsinki is gratefully acknowledged.

## BIOSKETCH

**Arun B. Mukherjee,** D.Sc., is a Research Scientist in the Department of Environmental Protection, University of Helsinki. He earned an M.Sc. degree in the field of Metallurgy from the Helsinki University of Technology and a D.Sc. degree from the Helsinki University in the field of Environmental Sciences. In his early career, Dr. Mukherjee worked as a metallurgist in a well known copper industry in Finland, India and Democratic Congo (formerly Zaire). In 1982, he started working in the field of environmental protection. His current research interests focus on the sources, fate, and effects of trace metals in the ecosystem. His research expertise includes: emission inventories of trace metals and chemicals, soil remediation, hazardous waste, waste management and recycling, groundwater arsenic problem, and greenhouse gases. He has conducted several national investigations in the field of trace metals and chemicals for the Ministry of Environment, Finnish Environment Institute, State Research Institute of Finland (VTT), University of Helsinki, and multinational companies. From time to time, Dr. Mukherjee has taught "Environmental Protection in the Developing Countries" for undergraduate students at Helsinki University of Technology. He has authored and co-authored 5 invited book chapters, and over 55 refereed publications in journals, conference proceedings, serials, and symposia.
